# La drépanocytose et le diabète: association rare chez un adolescent de race noire à Lubumbashi/ République Démocratique du Congo

**DOI:** 10.11604/pamj.2014.18.74.4589

**Published:** 2014-05-23

**Authors:** Léon Kabamba Ngombe, Augustin Mutombo Mulangu, Toni Lubala Kasole, Oscar Luboya Numbi

**Affiliations:** 1 Université de Kamina, Faculté de Médecine, Département de Santé Publique, Unité de Toxicologie, Kamina, République Démocratique du Congo; 2 Université de Lubumbashi, Faculté de Médecine, Département de Pédiatrie, Lubumbashi, République Démocratique du Congo

**Keywords:** Drépanocytose, diabète de type 1, adolescent, Sickle cell disease, type 1 diabetes, teenager

## Abstract

Les auteurs rapportent un cas d'un adolescent congolais de sexe masculin, âgé de 18 ans présentant une association rare de deux maladies génétiques à savoir la drépanocytose et le diabète, afin d'inviter le monde scientifique à mettre en marche des études moléculaires et génétiques approfondies pour appréhender ce phénomène rare.

## Introduction

Par leur fréquences et gravites, La drépanocytose et le diabète constituent des problèmes majeurs de santé publique en Afrique et dans le monde selon l'OMS. La drépanocytose est une affection génétique, caractérisée par la présence d'une hémoglobine anormale appelée Hémoglobine S. Elle est probablement la première maladie génétique dans le monde [1], et on estime que 50 millions d'individus sont atteints dans le monde [2]. La génétique intervient également dans la survenue du diabète de type 1 (diabète juvénile) [3]. La prévalence de ces deux pathologies varient d'un pays à l'autre. Nous rapportons une association rare entre la drépanocytose et le diabète chez un adolescent de race noire qui n'est pas assez décrit dans la littérature.

## Patient et observation

Le patient était de sexe masculin, âgé de 18 ans avec un poids de 67 Kg. Son état nutritionnel était bon (IMC 22Kg/m2, soit normal). A l'admission le motif de consultation était des douleurs articulaires atroces, ayant débuté 5 jours avant la consultation accompagnée d'une fièvre d'allure vesperonocturne. Dans les antécédents, l'adolescent est issu d'un mariage non consanguin avec une notion de drépanocytose dans la famille du père et de diabète dans la famille de la mère. L'adolescent est drépanocytaire connu depuis l’âge de 5 ans et diabétique connu sous insulinothérapie depuis 4 ans. Le diagnostic de la drépanocytose homozygote a été confirmé par l’électrophorèse des protéines qui a révèle la présence d'hémoglobine S. L'examen physique est sans particularité et les paramètres anthropométriques sont dans les normes: périmètre brachial 26 cm, périmètre abdominal 77 cm. Au vue des signes présentés par le patient, nous avons pensé à un paludisme et à une crise vaso-occlussive sur terrain de drépanocytose et de diabète de type 1. Les examens de laboratoire ont révélés une goutte épaisse positive, Hb: 11g/dl, le GB: 8000 mm3, VS: 10 mm/h, glycémie: 180 mg/dl. Un traitement fait des antalgiques, d'un antipaludéen, d'une réhydratation et d'un réajustement de son insulinothérapie avait été instauré. L'adolescent est régulièrement suivi pour la drépanocytose et le diabète dans nos services.

## Discussion

La drépanocytose est une maladie à caractère génétique, sa transmission se fait sur un mode autosomique dominant. Elle semble récessive puisque seuls les homozygotes sont gravement malades. De nos jours, il est connu que dans le diabète de type 1, le processus immunitaire destructeur est influencé par les gènes qui contrôlent le système immunitaire [3, 4]. Cette association de deux maladies à caractère génétique chez un adolescent de race noire attire notre attention; car dans la littérature, on retrouve de cas des thalassémies majeures avec diabète suite à des taux élevés du fer après plusieurs transfusions et traitement chélateurs [5, 6, 7]. Ceci n'est pas le cas dans notre observation. Des associations fortuites ou rares de la drépanocytose avec d'autres pathologies génétiques existent. C'est le cas de la drépanocytose et l'hypercholestérolémie familiale homozygote mentionnée par El Moussaif au Maroc [8]. A ce jour, la description de l'association entre la drépanocytose et le diabète dans la race noire est très rare dans la littérature scientifique de notre pays. Compte tenu de la prise en charge difficile et limitée de la drépanocytose ainsi du diabète dans nos milieux sous équipés et sous-développés; il s'avère important de mentionner cette rare association, afin d'inviter le monde scientifique à mettre en marche des études moléculaires et génétiques approfondies pour appréhender ce phénomène rare.

## Conclusion

L'association rare de la drépanocytose et du diabète demeure un domaine qui nécessite des recherches de la part du monde scientifique afin de comprendre cette dernière et d'améliorer la prise en charge des patients drépanocytaires.

## References

[1] Association drepavie. http://www.drepavie.org.

[2] Arnal C, Girot R (2002). Drépanocytose chez l'adulte. Encycl Méd Chir.

[2] Redondo MJ, Eisenbarth GS (2002). Genetic control of autoimmunity in type 1 diabetes and associated disorders. Diabetologia.

[4] Ueda H, Howson JMM, Esposito L, Heward J, Snook H, Chamberlain G (2003). Association of the T-cell regulatory gene CTLA-4 with susceptibility to autoimmune disease. Nature..

[5] Lanzkowsky PH (2000). Manual of pediatric hematology and oncology.

[6] Malik S, Syed S, Ahmed N (2009). Complications in transfusion-dependent patients of B-thalassemia major. Pak J Med Sci..

[7] Jahantigh M, Naderi M, Dorgalaleh A, Tabibian S (2014). Prevalence of diabetes and impaired glucose tolerance test in patients with thalassemia major. Zahedan J Res Med Sci (ZJRMS).

[8] Moussaif N El, Haddad N El, Iraqi H (2011). Hypercholestérolémie familiale homozygote et drépanocytose: association fortuite ou transmission génétique commune à propos d'un cas. Diabetes et Metabolism..

